# 174. Generic Lenacapavir HIV Pre-Exposure Prophylaxis could be Produced for $25 Per Person Per Year

**DOI:** 10.1093/ofid/ofaf695.004

**Published:** 2026-01-11

**Authors:** Cassandra Fairhead, Joseph Fortunak, Jevon Layne, Madison Johnson, Samyah Smalley, Andrew Lutterodt, David Roberts, Endalkachew Tadesse, Jasmine Lu, Eloan Pinheiro, Messay Wolde-Mariam, Andrew Hill, Toby Pepperrel

**Affiliations:** Royal Free Hospital, London, London, England, United Kingdom; Department of Chemistry, Howard University, Washington, D.C., USA 20905, Washington DC, District of Columbia; Johns Hopkins School of Medicine, 1650 Orleans St, RM162A, Baltimore, MDUSA 21287, Baltimore, Maryland; Department of Chemistry, Howard University, Washington, D.C., USA 20905, Washington DC, District of Columbia; Department of Chemistry, Howard University, Washington, D.C., USA 20905, Washington DC, District of Columbia; QIAGEN, Frederick MDUSA 20874, Frederick, Maryland; Department of Chemistry, Howard University, Washington, D.C., USA 20905, Washington DC, District of Columbia; Department of Chemistry, Howard University, Washington, D.C., USA 20905, Washington DC, District of Columbia; Lu Global Consulting, South Brunswick NJ, USA 08824, South Brunswick, New Jersey; Consultant, ARV development and manufacturing, Rio de Janeiro, Brazil 22753-806, Rio de Janeiro, Rio de Janeiro, Brazil; Pharmaceutical Industry Development Sector, Armauer Hansen Research Institute, Addis Ababa, Ethiopia, Addis Ababa, Adis Abeba, Ethiopia; University of Liverpool, London, England, United Kingdom; Biological and Environmental Sciences, University of Stirling, Stirling, Scotland, Stirling, Scotland, United Kingdom

## Abstract

**Background:**

1.3 million people acquired HIV in 2023, far exceeding UNAIDS targets. Affordable prevention access is essential, particularly given recent aid cuts. Six-monthly lenacapavir pre-exposure prophylaxis reduces transmission to almost zero. Lenacapavir’s price is a critical determinant of access. Previously we estimated generic lenacapavir could be produced for $41 per person-year. Following recent licencing and manufacturing advances an updated analysis is urgently warranted. We therefore update these estimates at meaningful volumes, and project global lenacapavir demand.

**Figure:**
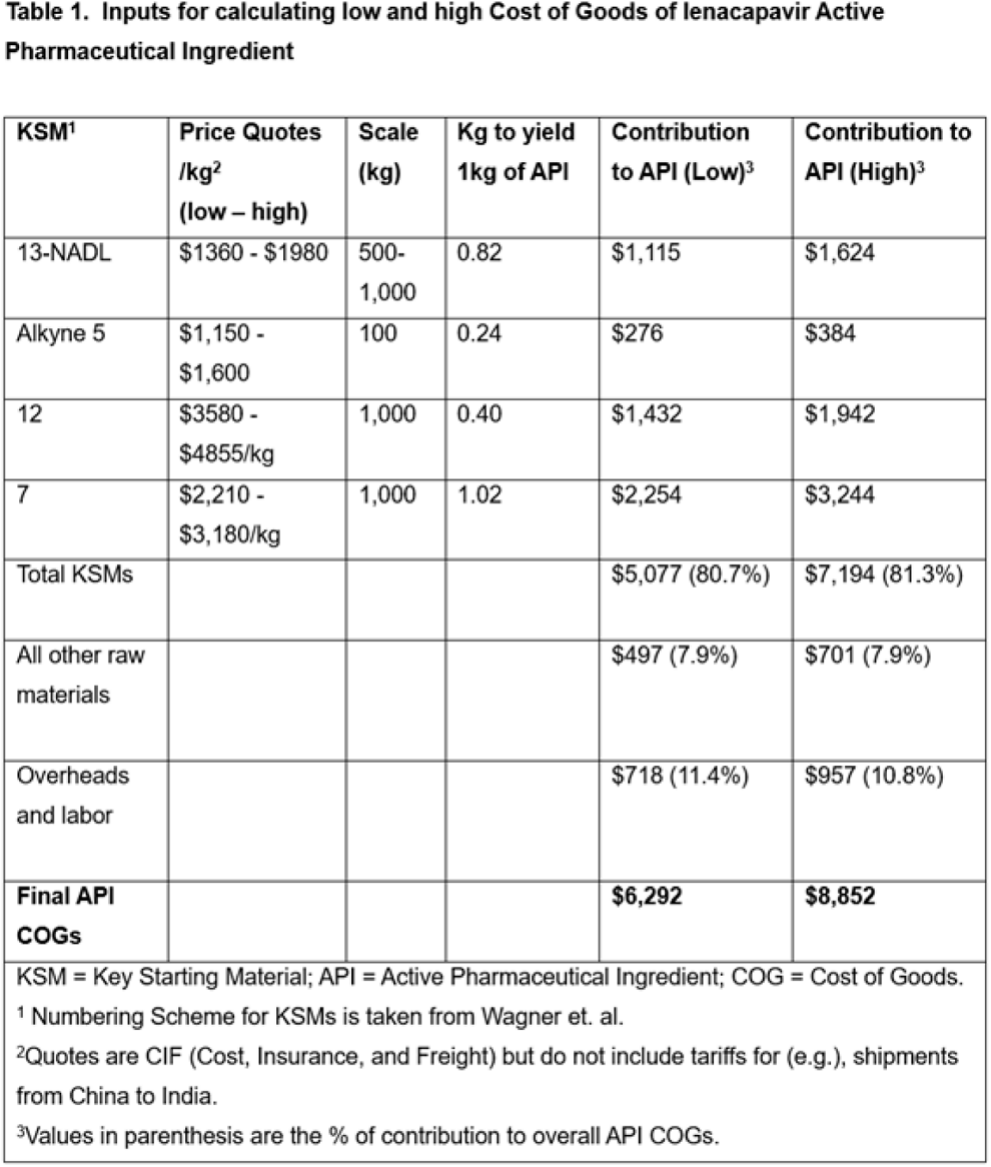

**Figure:**
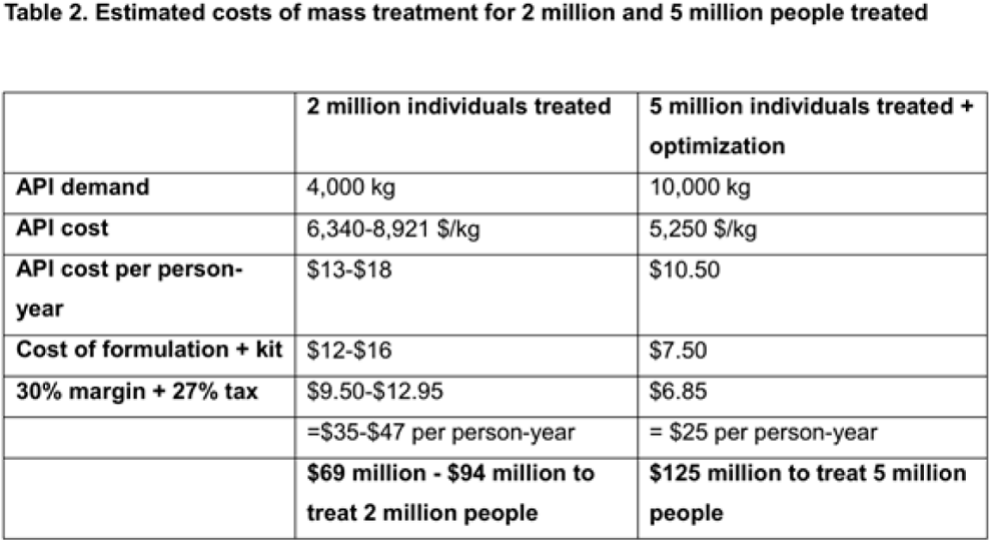

**Methods:**

June 2025 lenacapavir Key Starting Materials (KSMs) prices were obtained from vendors. Active Pharmaceutical Ingredient (API) Cost-of-Goods (COGs) from KSMs was projected using the commercial route of synthesis (ROS), assuming ≤2 API producers. Finished Pharmaceutical Product (FPP) Cost-plus steady-state model pricing for 2-5 million treatment-years was projected including formulation, labour, 30% profit and 27% taxation. Lenacapavir’s number-needed-to-treat in the PURPOSE-1/2 trials was multiplied by annual HIV acquisitions (1.3 million) to project demand.

**Figure:**
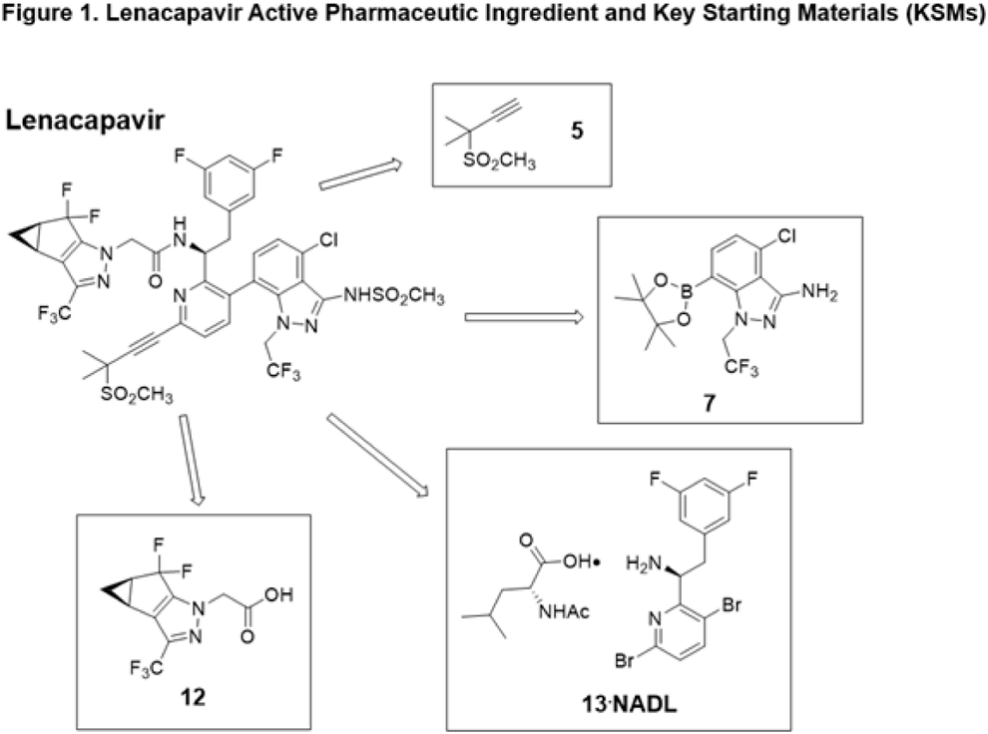

**Figure:**
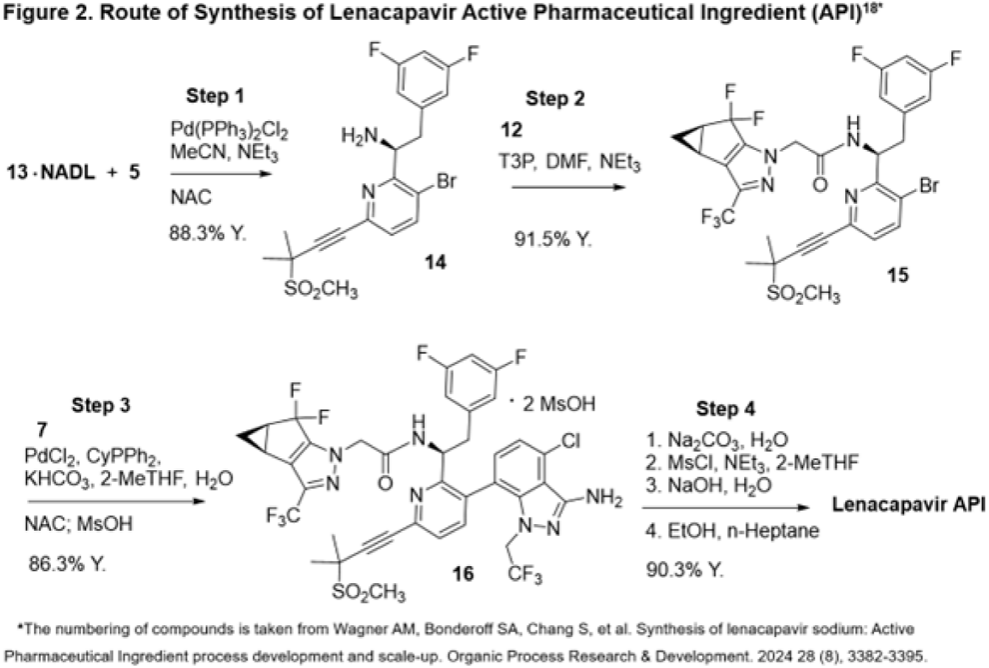

**Results:**

Lenacapavir KSM costs (Figure 1) have greatly decreased (Table 1) and a novel efficient ROS (Figure 2) is available. An API COGs between $6,340-$8,921/kg is achievable for a committed demand of two million treatment-years (4,000 kg/year of API). With Cost-plus pricing, generic lenacapavir FPP is projected to cost $35-$46 per treatment-year, reducing to $25 per treatment-year for five million people with expected modest manufacturing improvement (Table 2). Based on PURPOSE-1/2 number-needed-to-treats of 41.5-42.2, projected lenacapavir demand is 54-55 million people per year. UNAIDS predicts 6 million additional HIV acquisitions due to aid cuts: demand could therefore be several hundred million treatment-years by 2030. The 2 million lenacapavir treatment-years Gilead have committed to providing addresses < 0.5% of this potential need.

**Conclusion:**

Generic lenacapavir could be priced lower than oral alternatives, and 1000 times less than Gilead’s launch price ($28,218). With funding and licencing to support global access, lenacapavir could be a highly cost-effective prevention intervention.

**Disclosures:**

All Authors: No reported disclosures

